# Does the community service environment affect the willingness of older adults people to socialize for older adults care?

**DOI:** 10.3389/fpubh.2024.1370808

**Published:** 2024-05-28

**Authors:** Denghui Huang, Ran Feng, Minxuan Shi

**Affiliations:** School of Public Administration, Hohai University, Nanjing, China

**Keywords:** community service, environment, socialized pension, willingness to support the older adults, older adults people

## Abstract

Population ageing is a significant trend in social development and will remain a fundamental national condition in China for the foreseeable future. Socialized older adults care has become a crucial strategy for China to address population ageing. However, the current levels of acceptance and willingness to seek socialized older adults care among older adults are relatively low. This study focuses on examining how the community environment and services influence older adults people’s willingness to engage in social activities related to older adults care. Using data from the China Longitudinal Ageing Social Survey (CLASS) (*N* = 9,657), this study investigated the impact of the community service environment on older adults people’s willingness to participate in social activities related to older adults care through a logit model. The study revealed that 9.15% of older adults (*N* = 9,657) in China are willing to engage in social activities related to older adults care. Furthermore, the individual characteristics and family support of older adults play a significant role in shaping their willingness to engage in older adults care. The provision of medical services, daily care services, and entertainment venues by the community positively influence older adults people’s willingness to participate in social activities related to older adults care (*p* < 0.01), whereas psychological counselling services have a negative impact (*p* < 0.01). The impact of community services on older adults people’s willingness to engage in social activities related to older adults care varies due to factors such as smoking and chronic diseases. These findings provide valuable insights for improving older adults’ social engagement in China.

## 1 Introduction

In recent decades, population ageing has emerged as a global phenomenon (1). While it is a cause for celebration, it also presents significant challenges (2). As the ageing situation becomes increasingly severe, countries worldwide are striving to strike a balance between societal and familial care for older adults people, aiming to provide high-quality care services with minimal social costs while promoting healthy ageing (3). China, home to the world’s largest and fastest-growing older adults population, has witnessed a significant increase in the prominence of older adults care issues. The ageing rate of the Chinese population is remarkably rapid, with the proportion of individuals aged 65 and above exceeding 200 million in 2021 accounting for 14.2% of the national population. This marked a 0.7% increase from the previous year, further deepening the degree of ageing and transitioning society into a moderately ageing society. Furthermore, factors such as the substantial and rapid growth of the older adults population, increased mobility among younger individuals, changes in family structure, and the separation of children and older adults individuals have contributed to the increased role a socialized older adults care model.

In the increasingly competitive market economy, the traditional family older adults care model is becoming less suitable due to the weakening of family care functions, economic burdens, fast-paced urban life, and weakened family relationships. The current state of older adults care is concerning, as the ageing population poses significant challenges to social and economic development. Older adults care is crucial not only for the well-being of older adults people themselves but also for the development of families and society as a whole. It has become a key issue in social and economic development and an integral part of China’s national strategy. To address these challenges, China has introduced a “home-based, community-based, and institution-supported older adults care service system” (4). This model aims to establish a comprehensive service network and support system to address the diverse needs of older adults people. Given China’s unique cultural and demographic characteristics, the older adults care system has gradually transitioned from a family-based model to a socialized model with distinct features. This specific model helps relieve the burden of family care and caters to the professional care requirements of older adults people. However, the current level of socialized older adults care in China faces limitations stemming from factors such as capital investment, operational mechanisms, and mindset (5). Challenges include deficiencies in scale, specialization, service mechanisms, and supply–demand equilibrium. Older adults care institutions lack robust infrastructure, resulting in high vacancy rates, and the outreach of community-based care remains limited. Moreover, despite the aim of the socialized older adults care system to compensate for the decline in family care functions, family-based care remains deeply rooted in Chinese culture and continues to be in high demand. The majority of older adults individuals prefer to stay within their families, with approximately only 10% showing a willingness to opt for institutional care (6). The family setting remains the preferred environment for older adults care, with family members being the primary caregivers for older adults people. The reluctance of older adults people to embrace socialized older adults care poses a barrier to the advancement of this model. In summary, there is a pressing need for ongoing improvement and advancement of the socialized older adults care model in China, considering the cultural values and preferences of older adults people while addressing infrastructure deficiencies, service mechanisms, and supply–demand challenges.

Socialized older adults care services adhere to the principles of diversifying care service providers, making services accessible to the public, utilizing various care methods, and professionalizing care teams. Among these principles, the community plays a crucial role as a vital link between families and society, serving as a platform for distributing and extending older adults care resources. It is considered a pivotal pathway and future direction for addressing the challenges of ageing, with on-site care being the preferred approach for older adults people (7, 8). With the continuous promotion of and improvement in community development in China, there is a focus on encourageing older adults individuals to engage with the community, avail themselves of daytime care and health guidance services, and participate in social activities. Strengthening the functions of the community establishes a solid support foundation for socialized older adults care. In the context of weakened family care functions, community-based older adults care aligns more closely with the preferences and habits of older adults individuals in China, making it a preferred method for socialized older adults care. When older adults individuals are no longer able to care for themselves or lack the necessary conditions for home-based care, institutional older adults care provides an alternative, and the resources of care institutions can also be shared with the community. The community serves as the primary setting for the daily lives of older adults individuals, and the community environment plays a crucial role in their health and well-being. Therefore, communities hold significant importance in older adults care. To further increase the positive impact of communities on older adults care, particularly in increasing the acceptance of socialized care, it is essential to understand how the community service environment influences the willingness of older adults individuals to engage in socialized care. This study aims to explore the relationship between the community service environment and the willingness of older adults individuals to embrace socialized care by examining the specific effects of various types of community service environments. By analysing this relationship, we can gain insights into the further development of socialized older adults care, particularly in terms of targeted provision of and improvement in the community service environment. Socialized older adults care services can better serve and assist older adults people, contributing to healthy ageing.

Therefore, based on a review of current academic research, this paper explores how the community service environment affects the willingness of older adults people to socialize for older adults care using 2020 survey data from the China Comprehensive Survey of Ageing Society (CLASS). Specifically, this study found through a literature review that the community environment has multiple impacts on the lives of older adults people, and there are many factors that affect community willingness to care for older adults people. Therefore, this study focused on the impact of the community service environment on older adults care willingness. While validating previous research regarding the roles of factors such as gender, urban–rural development, education level, and age, this study utilizes the binary feature of older adults care willingness to analyse the specific impact of various aspects of the community service environment on older adults care willingness through logit model analysis and briefly explains the reasons for these impacts. By analysing the relationship between the two, we can provide ideas for further development of socialized older adults care, especially targeted provision of and improvement in the community service environment, so that socialized older adults care services can better serve and assist older adults people and thus achieve healthy ageing.

## 2 Literature review

Currently, there is significant academic interest in studying the impact of the community environment on the well-being of older adults people. The environment plays a vital role in supporting individuals throughout their lives by providing necessary resources (9). Additionally, there is a reciprocal relationship between individuals and their environments (10, 11). Research conducted by the World Health Organization, examining numerous older adults-friendly cities and communities globally, has shown that a favourable community living environment can mitigate the effects of life stress by offering social resources, thereby promoting healthy and positive ageing (9). First, the physical environment of the community significantly impacts the health of older adults people. Studies have demonstrated that the physical decline and loss of daily activity among older adults people are crucial considerations in promoting public health within the built environment (12). Housing accessibility is closely linked to older adults people’s ability to perform daily tasks (13). Therefore, it is important to develop personalized, age-friendly, and liveable spatial environments that facilitate healthy and positive ageing (14). Urban green spaces are particularly important for the well-being of older adults people because they provide opportunities for physical activity and social interactions (15). Moreover, environmental exposures can pose health risks to older adults people, who are considered vulnerable (16). Efforts to reduce pollution and improve socioeconomic conditions can significantly increase the health and longevity of older adults people (17). Consequently, incorporating environmental sustainability into the development of older adults living environments has garnered considerable attention (14). The community environment also influences the psychological well-being of older adults people. Scholars have explored the relationship between older adults-friendly environments and feelings of loneliness (18). Family environments for older adults people can sometimes be associated with negative experiences such as isolation and loneliness (7). There is a significant correlation between the dependency of older adults people and the availability of environmental resources within the community (19).

Second, regarding the community environment. Many communities in which older adults people reside are not designed to adequately meet their needs (20). However, a conducive environment can effectively address the requirements of older adults people. For instance, outdoor public spaces play a role in fostering and maintaining social connections among older adults community residents, thus fulfilling their social needs (21). Older adults individuals often have specific demands for the communities in which they live, particularly regarding the built environment and access to medical services (22). Therefore, when creating an older adults-friendly environment, priority is often given to the built environment, which includes factors such as accessible public transportation and housing, affordable and accessible healthcare services, and outdoor spaces and buildings that cater to the needs of older adults individuals. These elements contribute to maintaining accessibility for older adults people (23). However, some studies have shown that the impact of the physical environment on the health of older adults individuals diminishes with age, while the influence of the interpersonal environment and social participation increases (24). Furthermore, research has demonstrated a correlation between the community environment and cognitive function in older adults people (25). Enriching environmental stimuli can help prevent or slow cognitive decline (26). Living in a favourable community environment is often associated with a lower likelihood of experiencing depression (27). The community environment also moderates the relationship between social support and health among older adults people (28). Finally, the community environment significantly impacts the quality of life and satisfaction of older adults people. The living environment has a profound effect on the daily lives of older adults people, even surpassing the impact of social interactions (29). Moreover, the entertainment and social environment within the community influence the life satisfaction of older adults people, with the neighbourhood environment positively correlated with life satisfaction (30). Higher levels of activity among older adults people are associated with increased quality of life and satisfaction (31). The neighbourhood environment plays a crucial role in creating older adults-friendly communities (32). Community health services directly affect the health status of older adults people. The level of medical care provided by community health services, combined with the community environment, plays a fundamental role in ensuring the health of older adults residents (33). Daily care services address the living needs of older adults people. Due to physical health decline and limited mobility, older adults individuals require care from others, with specific variations based on their personal health status and family circumstances (34). Some older adults individuals require long-term care (35), while others need assistance with daily activities (36). Additionally, external interventions can help alleviate the burden on older adults families (37). Therefore, the community service environment can influence the preferences of older adults people for care services (8) and improve their overall quality of life (38).

Numerous studies have explored the factors influencing the willingness of older adults individuals to receive care. Family-based older adults care remains the primary choice, while socialized care options such as community-based and institutional care still have significant room for development (6). The willingness of older adults individuals to receive care is influenced by various factors, and extensive research has been conducted to understand how these factors impact their decisions. First, economic factors play a significant role in shaping the willingness of older adults individuals to receive care. For instance, research has shown that care arrangements for older adults people in China are influenced by the balance of family care resources, economic considerations, and social status (39). The economic self-assessment of individuals affects their willingness to receive care later in life (40). Most low-income older adults individuals (80.4%) prefer to receive care at home, even if their health and functional abilities have declined and they are unable to live independently (41). Rural residents generally express a willingness to pay for socialized older adults care services in rural areas, but their own economic conditions may limit their choices (42). Older adults individuals who perceive service fees to be high are more inclined to stay at home due to the high cost of institutional care (43). The economic status of the family (39) and participation in urban resident basic medical insurance (44) also influence the willingness of older adults individuals to reside in institutional care. This suggests that older adults individuals with sufficient economic resources may be more likely to choose nursing homes or other forms of long-term care (44). Conversely, those with limited economic resources may rely more on family care or choose to age in place at home. Second, social support and family relationships have a significant impact on the willingness of older adults individuals to receive care (45). The presence of close family relationships (46), support from children or other relatives (47), living arrangements with spouses and children (48, 49), proximity to children (50), community support and services (51), interpersonal relationships, and spiritual needs (52) can influence individuals’ willingness to receive care. Additionally, the individual characteristics of older adults people also play a role in their willingness to receive care. Some studies indicate that marital status has a positive impact on the acceptance of socialized older adults care (42). However, other studies suggest that widowed older adults individuals may be more inclined to choose institutional care than married older adults individuals (43). Moreover, factors such as gender (40), age (45), rural or urban residence (53), education level (39, 47), and occupation (54) also influence the willingness of older adults individuals to receive care. Studies have shown that older adults individuals in urban areas of China are more likely to opt for nursing home services or prefer government-provided care than those in rural areas (55). Additionally, the personal health status and functional abilities of older adults people are important factors in their willingness to receive care. Self-care abilities and health status have a negative impact, with nondisabled older adults individuals showing significantly less willingness to receive institutional care than those with disabilities (56, 57). The presence of chronic health conditions also influences the choice of older adults care options (11).

In summary, research on the impact of the community environment on older adults individuals has focused primarily on the effects of the community-building environment or the ecological environment on the health of older adults people (14, 16, 58), their cognitive abilities (25, 26), their mental health (27), their life satisfaction (32, 59), and their overall perceived convenience of life (13, 60). While there is relatively little research on the impact of the community environment on older adults care willingness, studies have explored individual sociodemographic characteristics, social support, and economic conditions concerning older adults people’s willingness to receive care (46, 47).

The community serves as a vital component in delivering the “last mile” of community home-based older adults care services and acts as the fundamental social unit for implementing national older adults care service policies. The community service environment is an essential foundation in the daily lives of older adults people (61). As a significant place for the daily routines of older adults people, the community profoundly influences their lives. The demand for socialized older adults care services in Chinese communities has evolved with social development, where the supply level and capacity of urban community public services serve as crucial indicators of urban community governance capacity (62). In response to ageing as a national strategy, China aims to increase the construction of community older adults care service systems, offering high-quality and diverse older adults care services from various entities within the community to meet the diverse service needs of the older adults population and increase their life satisfaction. However, while actively developing older adults care services, it is essential to assess whether providing multiple types of high-quality socialized older adults care services affects older adults people’s willingness to provide care, particularly regarding the potential impact of the community service environment. Understanding these dynamics can help optimize policy design, promote improvements in the community service environment, and increase the acceptance of socialized older adults care among the older adults population. Therefore, this article utilizes publicly available survey data to investigate how the community service environment influences the willingness of older adults individuals to engage in socialized older adults care, aiming to offer insights for refining policy design, advancing the improvement in the community service environment, and boosting the acceptance of socialized older adults care among the older adults population. Therefore, this paper utilizes publicly available survey data to validate relevant research on the factors influencing older adults care willingness and investigates how the community service environment impacts the socialization of older adults care willingness. By referencing the specific aspects of the social service environment highlighted in the literature, selecting pertinent research variables and suitable analytical models, and leveraging the outcomes of the model analysis to elucidate how the community service environment influences the willingness of older adults individuals to engage in socialized older adults care, along with providing potential explanations for these effects, this study offers insights for refining policy, further improving the community service environment, and bolstering the acceptance of socialized older adults care among older adults people.

## 3 Data sources and methods

### 3.1 Data sources

The data utilized in this study are derived from the 2020 survey data of China’s CLASS. The CLASS is a national and ongoing large-scale social survey initiative. By systematically gathering data on the social and economic circumstances of the older adults population in China, the project aims to comprehend the diverse issues and obstacles encountered by older adults people during the ageing process and assess the tangible impacts of various social policy measures in improving their quality of life. The CLASS project initiated two pilot surveys in 2011 and 2012, with the inaugural nationwide baseline survey conducted in 2014. Subsequently, the survey was conducted biennially. The CLASS relies on the China Social Survey Network (CSSN) to oversee the entire field data collection process.

It employs a stratified and multistage probability sampling approach, selecting county-level areas (comprising counties, county-level cities, and districts) as primary sampling units (PSUs) and village/neighbourhood committees as secondary sampling units (SSUs). The survey targets Chinese citizens aged 60 and above, employing a sampling method that involves drawing samples from each village/neighbourhood committee. During field investigations, local supervisors lead visiting teams to complete questionnaire visits at various PSUs. Using the address of each SSU as a sampling box, households are selected, and indoor sampling of older adults individuals aged 60 and above is conducted, with one interviewee selected for the interview. Face-to-face visits are conducted for the survey. Additionally, a community questionnaire is administered in each surveyed community. To ensure data quality, on-site supervision, remote data analysis, and telephone verification are employed for quality control throughout the investigation process.

The data collected by the project are shared with the academic community free of charge after cleaning, weight calculation, and documentation writing, offering high-quality data support for academic research and policy development in various fields related to older adults people. The questionnaire design is scientific and systematic, and the data obtained exhibit strong representativeness and robustness, rendering it a valuable resource for numerous scholars studying the older adults population in China. The survey focuses on individuals aged 60 and above across 28 provinces, cities, and autonomous regions in China, with an initial sample size of 11,398 individuals. After excluding missing values for relevant variables, the final sample for this study comprised 9,657 individuals.

### 3.2 Variable selection and explanation

The specific meanings and coding assignments of each variable selected for this study are provided in Table 1.

**Table 1:** Variable assignments and explanations.

Variable	Variable description
Address	Rural = 0, non-rural = 1
Gender	Male = 1, female = 0
Age	Age of the older adults
Education level	“Illiteracy,” “private school/literacy class,” “primary school,” “junior high school,” “high school / technical secondary school,” “junior college” and “bachelor degree or above” are assigned to 0, 3, 6, 9, 12, 15 and 16 in turn
Marital status	In marriage = 1, others = 0
Residential arrangements	Living with family = 1, living alone 0
Self-rated health	“Very unhealthy,” “relatively unhealthy,” “average,” “relatively healthy” and “very healthy” are assigned as 1, 2, 3, 4 and 5 respectively
Self-care situation	Self-care = 0, unable to take care of oneself = 1
Chronic disease	Yes = 1, No = 0
Smoking	Smoking = 1, non-smoking = 0
Ln_income	Logarithm of annual income of the older adults
Religious belief	Yes = 1, None = 0
Medical Service	“Yes” = 1 and “No” = 0 for each medical service, and the scores of 7 services are added
Daily life services	“Yes” = 1 and “No” = 0 for each daily life service, and the scores of 5 services are added
Psychological counselling service	“Yes” = 1 and “No” = 0 for each psychological counselling service, and the scores of the four services are added
Place of entertainment	Is there a place for entertainment, “yes” = 1, “no” = 0

The dependent variable in this study is the willingness of older adults people to receive care. The questionnaire asked, “Where do you plan to primarily receive care as you age? The individuals who answered “community day care stations or nursing homes” and “nursing homes” were categorized as opting for socialized older adults care and assigned a value of 1. Those who answered “their own home” or “children’s home” were categorized as opting for family older adults care and assigned a value of 0.

The core explanatory variable in this study is the community service environment. Existing research has defined and categorized the community service environment based on different levels or dimensions, such as the nature of the service content or the functional division of community service projects, which are aligned with the needs of community older adults care services (38). Drawing on this research (10), the community service environment is subdivided into four categories: medical services, daily life care, psychological counselling services, and entertainment venues. First, medical services provision healthcare and related services to the community. The questionnaire item “Have you utilized community medical institutions (health service centres, clinics, etc.) in the past 12 months?” had values assigned for “home care,” “home visits,” “rental of rehabilitation aids,” “rehabilitation training,” “free physical examinations,” “establishment of health records,” and “health lectures.” Responses of “Yes” were assigned a value of 1, while responses of “No” were assigned a value of 0. The scores for the seven services were then summed to obtain the medical service score. Higher scores indicate greater availability of medical services in the community. Second, daily life care comprises the assistance and support provided by the community to older adults people in their daily activities. The questionnaire item “Does your community provide the following services?” had values assigned based on the provision of “accompanying patients,” “assistance with daily shopping,” “household chores assistance,” “older adults dining tables or food delivery,” and “daycare stations or nursing homes.” Responses of “Yes” were assigned a value of 1, while responses of “No” were assigned a value of 0. The values for the five services were then summed to obtain the daily life services score. Psychological counselling services comprise the support and guidance provided by the community to older adults people in terms of psychological counselling and relief. The questionnaire item “Does your community provide the following services?” had values assigned based on the availability of “door-to-door visits,” “older adults service hotlines,” “legal aid,” and “psychological counselling.” Responses of “Yes” were assigned a value of 1, while responses of “No” were assigned a value of 0. The values for the four services were then summed to obtain the psychological counselling services score. Finally, entertainment venues are the facilities and spaces provided by the community for older adults individuals to engage in physical activities, socialize, and learn. Answers to the questionnaire item “Does your community have the following activity venues or facilities?” were assigned a value of 1 if at least one venue was available and a value of 0 if no venues were available. The score for entertainment activity venues is derived accordingly.

We used control variables and referred to existing research (50) to further control for other factors that affect older adults people’s willingness to accept care, namely, personal characteristics, family level, and health level factors. These variables include age, gender (male = 1, female = 0), and education level (illiterate,” “private school/literacy class,” “primary school,” “junior high school,” “high school vocational school,” “college” and “undergraduate and above” are assigned values of 0, 3, 6, 9, 12, 15, and 16, respectively), ethnicity (ethnic minority = 0, Han = 1), marital status (in marriage = 1, others = 0), self-care ability (self-care = 1, cannot self-care = 0), urban–rural status (rural = 0, nonrural = 1), living arrangement (living with family = 1, living alone = 0), religious belief (yes = 1, no = 0), self-rated health (“very unhealthy,” “relatively unhealthy,” “average,” “relatively healthy,” and “very healthy” are assigned values of 1, 2, 3, 4, and 5, respectively), chronic diseases (presence = 1, absence = 0), smoking status (smoking = 1, nonsmoking = 0), and logarithmic treatment of individual annual income for older adults individuals.

### 3.3 Analysis methods

The dependent variable in this study is the willingness to receive social older adults care, which is a binary variable categorized into two groups: not selecting social older adults care (assigned a value of 0) and choosing social older adults care (assigned a value of 1). The data analysis was conducted using STATA 17.0 statistical software, primarily employing binary logistic regression analysis to establish a model and examine the relationships among variables.

In the binary logistic model, the dependent variable is the probability $P\left(Y=1|x_{i}\right)$ (hereinafter referred to as *p*) of choosing socialized older adults care. To determine the nonlinear relationship between *p* and the linear regression equation composed of the explanatory variable, $\beta_{0}+\overset{k}{\underset{i=1}{\sum }}\beta_{i}x_{i}$ , $\Omega =\frac{p}{1-p}$ is constructed as the odds ratio, which represents the ratio of the probability of choosing socialized older adults care willingness to the probability of not choosing socialized older adults care willingness. To control this value range between $\left(-\infty ,+\infty \right)$ , the logarithm of Ω is taken. A multiple factor binary logistic regression model is obtained, expressed in Eq. 1:

(1) $$\log\mathrm{it}\left(p\right)=\ln\frac{p}{1-p}=\beta_{0}+\overset{k}{\underset{i=1}{\sum }}\beta_{i}x_{i}$$

where *p* is the dependent variable, representing the probability of residents choosing socialized older adults care; *k* represents the number of variables; $x_{i}$ is the explanatory variable, including the core explanatory variable and control variable composed of community medical services, daily life services, psychological counselling, and entertainment venues; $\beta_{0}$ is a constant term; and $\beta_{i}$ is the parameter to be estimated.

We logarithmize Eq. 1 to obtain Eq. 2:

(2) $$\frac{p}{1-p}=e^{\beta_{0}+\overset{k}{\underset{i=1}{\sum }}\beta_{i}x_{i}}$$

Finally, we obtained Eq. 3.

(3) $$p=\frac{1}{1+e^{-\left(\beta_{0}+\overset{k}{\underset{i=1}{\sum }}\beta_{i}x_{i}\right)}}$$

At this point, the modelling is completed.

## 4 Results

### 4.1 Survey sample overview

The basic information of the sample is presented in Table 2. In terms of the gender structure of the survey subjects, there were 4,854 male participants, accounting for 50.26% of the sample, and 4,803 female participants, accounting for 49.74%. The gender distribution was relatively balanced. Regarding the urban–rural divide, 45.62% of the respondents were from rural areas, while 54.38% were from nonrural areas. In terms of educational level, 2,328 older adults individuals were illiterate, accounting for 24.11% of the sample. Approximately 61% of the participants had received primary and secondary education, and 10.4% had a high school education or above. Only 5.18% of the older adults participants had clear religious beliefs, while 94.82% did not have specific religious beliefs. Among the participants, 6,910 older adults individuals did not smoke, accounting for 71.55% of the sample, while 28.45% of the older adults participants smoked. In terms of self-care, 93.65% of older adults individuals were capable of taking care of themselves, while 6.35% required assistance from family or others for daily activities. Regarding chronic diseases, a relatively high proportion of older adults individuals (78.60%) suffered from chronic diseases, while 21.40% did not have any chronic diseases.

**Table 2:** Descriptive statistics of sample variables (*N* = 9,657).

Characteristic	Frequency	Relative frequency (%)
**Willingness**		
Social pension	884	9.15
Family pension	8,773	90.85
**Gender**		
Female	4,803	49.74
Male	4,854	50.26
**Address**		
Rural	4,406	45.62
Non-rural	5,251	54.38
**Education level**		
Illiteracy	2,328	24.11
Private school/literacy class	395	4.09
Primary school	3,570	36.97
Junior high school	2,359	24.43
High school /technical secondary school	802	8.30
Junior college	164	1.70
Bachelor degree or above	39	0.40
**Self-care situation**		
Self-care	9,044	93.65
Unable to take care of oneself	613	6.35
**Chronic disease**		
No	2067	21.40
Yes	7,590	78.60
**Smoking**		
Yes	2,747	28.45
No	6,910	71.55
**Religious belief**		
No	9,157	94.82
Yes	500	5.18

Characteristic	Mean	Sd
ln_income	71.65	6.607
age	3.529	4.382

In terms of the overall willingness of the survey participants to provide older adults care, the inclination towards socialized older adults care methods was relatively low, with only 9.15% of older adults individuals expressing a willingness to choose such options. Most older adults individuals still preferred family-based older adults care. This indicates that the willingness of older adults individuals in China to opt for socialized older adults care is not particularly high. Therefore, it is crucial to identify the factors that influence their willingness, particularly whether the community service environment significantly affects their inclination to seek older adults care.

### 4.2 Model analysis

This article aims to analyse the impact of the community service environment on the willingness of older adults people to engage in social activities for older adults care. To achieve this, a model is constructed to examine the effects of the control variables and independent variables on the dependent variable. The results of this analysis are presented in Table 3.

**Table 3:** Logit model analysis of community service environment on the willingness of the older adults to provide for the aged socially.

	Model (1)	Model (2)	Model (3)	Model (4)	Model (5)
Medical Service		0.454^***^ (0.076)	0.436^***^ (0.077)	0.228^***^ (0.040)	0.036^***^ (0.007)
Daily life services		0.545^***^ (0.040)	0.496^***^ (0.040)	0.284^***^ (0.022)	0.071^***^ (0.005)
Psychological counselling service		−0.176^***^ (0.051)	−0.214^***^ (0.049)	−0.120^***^ (0.026)	−0.023^***^ (0.005)
Place of entertainment		1.035^***^ (0.117)	0.787^***^ (0.123)	0.354^***^ (0.055)	0.036^***^ (0.005)
Address	0.056 (0.108)		−0.063 (0.104)	−0.011 (0.051)	−0.007 (0.007)
Gender	0.059 (0.087)		0.045 (0.091)	0.025 (0.046)	0.003 (0.007)
Age	−0.023^***^ (0.006)		−0.020^***^ (0.006)	−0.011^***^ (0.003)	−0.002^***^ (0.000)
Education level	0.047^***^(0.011)		0.041^***^(0.011)	0.021^***^ (0.006)	0.003^***^ (0.001)
Marital status	−0.142 (0.109)		−0.151 (0.114)	−0.085 (0.057)	−0.013 (0.008)
Residential arrangements	−0.477^***^ (0.134)		−0.605^***^ (0.139)	−0.304^***^ (0.071)	−0.044^***^ (0.011)
Self-rated health	−0.127^***^ (0.046)		−0.047 (0.047)	−0.018 (0.024)	−0.003 (0.003)
Self-care situation	0.416^***^(0.143)		0.387^**^ (0.151)	0.214^***^ (0.078)	0.029^**^ (0.013)
Chronic disease	0.671^***^ (0.111)		0.404^***^ (0.115)	0.185^***^ (0.056)	0.022^***^ (0.006)
Smoking	−0.244^**^ (0.095)		−0.238^**^ (0.101)	−0.110^**^ (0.051)	−0.018^**^ (0.007)
Ln_income	0.123^***^ (0.012)		0.081^***^ (0.012)	0.042^***^ (0.006)	0.006^***^ (0.001)
Religious belief	0.024 (0.178)		−0.040 (0.192)	0.002 (0.095)	0.002 (0.012)
_cons	−1.292^***^ (0.496)	−3.586^***^ (0.113)	−2.040^***^ (0.530)	−1.105^***^ (0.269)	0.156^***^ (0.038)
N	9,657	9,657	9,657	9,657	9,657
R^2^	0.077	0.111	0.142	0.144	0.108

Standard errors in parentheses *p < 0.1, **p < 0.05, ***p < 0.01.

Studies have extensively examined the factors influencing the retirement preferences of older adults individuals. In this study, Model 1 incorporates several control variables, including urban and rural areas, gender, age, and education level, to investigate their impact on the willingness of older adults individuals to engage in social activities for older adults care. The findings reveal that certain control variables significantly influenced this willingness. Specifically, age exhibited a significant negative association with the willingness of older adults individuals to participate in socialized older adults care. This suggests that older individuals are less inclined to accept socialized older adults care and are more likely to prefer family-based care. On the other hand, the level of education and personal annual income have a positive effect on the willingness of older adults individuals to engage in socialized older adults care. Higher education levels and personal income corresponded to a greater willingness to accept socialized older adults care. Furthermore, residential arrangements, self-rated health, and self-care abilities exhibited a significant negative impact on the willingness of older adults individuals to participate in social activities for older adults care. Older adults individuals who did not live alone were less willing to embrace socialized older adults care, and those who perceived their health as better also showed a reduced willingness. Conversely, the presence of chronic diseases positively influenced the willingness of older adults individuals to engage in socialized older adults care, indicating that individuals with chronic illnesses are more inclined to accept this form of care. Moreover, older adults individuals who smoked displayed an even lower willingness to participate in socialized older adults care. In contrast, factors such as gender, urban and rural residency, marital status, and religious beliefs did not significantly affect the willingness of older adults individuals to engage in social activities for older adults care.

Model 2 incorporates four key independent variables related to the community service environment, namely, medical services, daily life services, psychological counselling services, and facility locations. The analysis results from Model 2 indicate that even without including control variables such as urban and rural residency and gender, medical services, daily life services, psychological counselling services, and entertainment venues significantly influenced the willingness of older adults individuals to engage in socialized older adults care, confirming the research hypothesis. Among these variables, medical services, daily life services, and entertainment venues exhibited a significant positive impact on the willingness of older adults people to accept socialized care. This finding implies that well-developed community medical services, daily life services, and facilities play crucial roles in increasing the willingness of older adults people to embrace socialized older adults care, which aligns with the initial research hypothesis. However, it is noteworthy that psychological counselling services decreased the willingness of older adults individuals to engage in socialized older adults care. In other words, increased utilization of psychological counselling services by older adults people was correlated with a decreased willingness to accept socialized older adults care, contradicting the original research hypothesis.

Model 3 incorporates both key independent variables and control variables for analysis. Compared to Model 1, Model 2 shows certain improvements, and the causal relationship between the community service environment and the willingness of older adults individuals to engage in socialized older adults care remained consistent with that of Model 2. The presence of medical services, daily life services, and entertainment venues within the community service environment significantly and positively influenced the willingness of older adults individuals to socialize for older adults care, in line with the research hypothesis. However, the utilization of psychological counselling services did not facilitate the acceptance of socialized older adults care by older adults people, contradicting the research hypothesis.

Models 4 and 5 were validated using probit and linear regression models, respectively, based on the findings from Model 3. The results from Models 4 and 5 are largely consistent with those from Model 3. The community service environment had a significant impact on the willingness of older adults individuals to engage in socialized older adults care, supporting the research hypothesis. Specifically, the presence of community medical services, daily life services, and entertainment venues positively influenced the willingness of older adults individuals to socialize for older adults care. However, psychological counselling services did not increase the willingness of older adults individuals to engage in socialized older adults care but rather had a significant negative impact, contrary to the research hypothesis.

### 4.3 Heterogeneity analysis

To explore potential variations in the impact of the community service environment on the willingness of older adults individuals to engage in social activities for older adults care, this section conducts a heterogeneity analysis based on factors such as smoking habits and chronic disease status. The older adults population was divided into groups according to these characteristics, and the differences in the impact of the community service environment on people’s willingness to socialize for older adults care were further examined. The results of this heterogeneity analysis are presented in Table 4.

**Table 4:** Estimated results of heterogeneity analysis.

	Model (6)	Model (7)	Model (8)	Model (9)
	Smoking	Non-smoking	No chronic diseases	Have chronic diseases
Medical service	0.468^***^ (0.151)	0.435^***^ (0.091)	0.240 (0.242)	0.463^***^ (0.082)
Daily life services	0.457^***^ (0.080)	0.506^***^ (0.046)	0.305^**^ (0.141)	0.509^***^ (0.041)
Psychological counselling service	−0.255^**^ (0.101)	−0.189^***^ (0.056)	−0.036 (0.156)	−0.229^***^ (0.052)
Place of entertainment	0.084 (0.200)	1.149^***^ (0.160)	0.574^**^ (0.264)	0.842^***^ (0.138)
_cons	−0.474 (1.038)	−2.718^***^ (0.610)	−2.690^*^ (1.506)	−1.662^***^ (0.542)
Control variable	Yes	Yes	Yes	Yes
*N*	2,747	6,910	2067	7,590

Standard errors in parentheses *p < 0.1, **p < 0.05, ***p < 0.01.

The estimation results of Models 6 and 7 indicate that the influence of medical services, daily care services, and psychological counselling services in the community service environment on the willingness of older adults individuals to engage in social activities for older adults care did not vary with smoking status. However, the impact of entertainment venues on the willingness of older adults individuals to participate in socialized older adults care was influenced by smoking status. Specifically, for nonsmoking older adults individuals, entertainment venues had a positive and promoting effect on their willingness to engage in social activities for older adults care. On the other hand, for smoking older adults individuals, the positive impact of entertainment venues on their willingness to participate in socialized older adults care was not statistically significant.

The estimation results from Models 8 and 9 indicate that the willingness of older adults individuals to engage in social activities for older adults care was not influenced by daily life services of entertainment venues in the community service environment. Moreover, there was no difference in the impact of these factors based on whether older adults individuals had chronic diseases, as both showed significant positive effects. However, there were variations in the impact of medical services and psychological counselling on the willingness of older adults individuals to engage in social activities for older adults care, depending on whether they had chronic diseases. Specifically, for older adults individuals with chronic diseases, community medical services had a significant positive impact on their willingness to participate in socialized older adults care. This suggests that the presence of quality medical services in the community encourages older adults individuals with chronic diseases to embrace socialized older adults care. On the other hand, this impact was not statistically significant for older adults individuals without chronic diseases. Similarly, for older adults individuals with chronic diseases, community psychological counselling had a significant negative impact on their willingness to engage in social activities for older adults care. This implies that psychological counselling services in the community make older adults individuals with chronic diseases less inclined to accept socialized older adults care. However, this impact was not significant for older adults individuals without chronic diseases.

## 5 Discussion

The willingness of older adults individuals to engage in social activities for older adults care is influenced by various factors, as supported by previous studies. This study validates the findings of previous research in several ways. First, the study confirms that the individual characteristics and the family environment of older adults people impact their willingness to participate in social activities for older adults care. Consistent with previous studies (45, 47), older individuals are more inclined towards family-based older adults care, resulting in a lower willingness to engage in socialized older adults care. Additionally, the positive effects of education level, personal annual income, and willingness to engage in social activities for older adults care among older adults people align with previous findings (39, 43, 47). Moreover, the study revealed that living arrangements, such as living with a spouse and children, significantly impact the willingness of older adults individuals to engage in social activities for older adults care, consistent with previous findings (48, 49). Family care and support from children (47) make individuals more inclined to prioritize family care. The health status of older adults people also significantly influences their willingness to engage in social activities for older adults care, with self-care ability showing a notable negative impact (57). Additionally, chronic diseases positively influence the willingness of older adults individuals to engage in social activities for older adults care (11). Older adults individuals with chronic diseases are more willing to accept socialized older adults care due to their increased need for care, particularly medical services. Furthermore, the study revealed that older adults individuals who smoke are less willing to accept socialized older adults care. This can be attributed to the fact that socialized older adults care may restrict their smoking habits, conflicting with their long-term smoking behaviours. As a result, they may resist external constraints on their lifestyle choices. However, the study did not find a significant impact of factors such as gender, urban or rural residency, or marital status on the willingness of older adults individuals to engage in social activities for older adults care. These results conflict with previous findings (40, 43, 55), possibly due to differences in sample selection.

The community service environment exerts a substantial influence on the willingness of older adults individuals to engage in socialized older adults care, confirming the research hypothesis. Retirement and physical limitations often confine older adults individuals to their homes and communities, making the community environment highly significant (63). The impact of the community environment on older adults people is multifaceted, particularly regarding the social service environment (54). Previous studies have demonstrated that a favourable community environment can mitigate the effects of life stress by providing social resources, thereby promoting healthy and positive ageing (9). This provides an explanation for the inclination of older adults individuals towards socialized older adults care. Older adults people have a desire for self-realization and enjoyment in their later years, and the community can provide support in fulfilling these needs. This study validates these findings.

First, the study revealed that the community medical service environment significantly increases the willingness of older adults individuals to engage in social activities for older adults care. As older adults individuals experience physical decline and multiple health issues, their demand for a suitable community medical service environment becomes essential (22). This explains why a favourable medical service environment in the community positively impacts the willingness of older adults individuals to engage in socialized older adults care. Access to quality medical services enables older adults people to maintain their dignity and quality of life, making socialized older adults care a more natural choice.

Second, the study highlights the significant impact of daily care services on the willingness of older adults individuals to engage in social activities for older adults care. Caring for an older adults family member is often described as challenging (64), and it can also hinder the daily lives of caregivers, particularly their mobility. In this context, the provision of daily care services by the community meets the life needs of older adults people, making them more willing to participate in socialized older adults care.

Third, community psychological counselling services is significantly and negatively correlated with the willingness of older adults individuals to engage in socialized older adults care. Several factors may explain the inconsistency between these results and the initial hypothesis. It is believed that while older adults-friendly environments can influence the psychology of older adults people (18), social support plays a crucial role in individuals’ mental well-being (65). Among various older adults care approaches, socialized older adults care is gradually gaining popularity. However, traditional beliefs deeply ingrained in Chinese older adults individuals often lead them to prefer family-based older adults care. This preference stems from the belief that external psychological comfort cannot replace the emotional satisfaction derived from familial relationships, particularly the spiritual care provided by spouses and children. First, within the familial older adults care setting, older adults individuals receive care from their spouses and children and experience the warmth of family affection. Given the profound emphasis on family values in China, older adults individuals aspire to spend their later years at home, surrounded by the care and love of their families. This emotional fulfilment cannot be provided by community psychological counselling services. Second, as their children pursue work opportunities, older adults people may lack continuous care and attention, potentially resulting in feelings of loneliness and emptiness. In such circumstances, they yearn for emotional support and psychological solace from their families, which community-based psychological counselling services may not adequately provide, failing to meet their innermost needs. Moreover, there is a notable disparity between the desire for familial care and the psychological counselling services offered by the community, intensifying their desire for emotional support and psychological comfort from their families. Concerns about potential alienation from family and friends, the erosion of existing social networks, and heightened feelings of loneliness may deter older adults individuals from opting for socialized older adults care, reinforcing their preference for family-based care. Cultural norms, the unique nature of spiritual solace provided by children, and apprehensions regarding future social interactions are key factors contributing to the limited efficacy of community psychological counselling services in fostering the willingness of older adults individuals to engage in socialized older adults care.

Fourth, the study revealed that community entertainment venues positively increase the willingness of older adults individuals to engage in social activities for older adults care. Age-friendly and liveable spatial environments contribute to healthy and positive ageing (14). Older adults individuals require physical venues that provide safe spaces for recreational activities and social interactions (15, 32). Entertainment venues facilitate the formation and maintenance of social relationships among older adults residents in the community (21), meeting their social needs. This positively impacts the health of older adults people (24), helps them maintain their cognitive abilities (26), reduces their rate of depression (27), and ultimately increases their life satisfaction (30, 31). The presence of community entertainment venues can fulfil the needs of older adults people and, to some extent, increase their willingness to engage in social activities for older adults care.

The impact of the community service environment on the willingness of older adults individuals to engage in social activities for older adults care varies across subgroups, such as those with chronic diseases and smokers. In China, there is a concerning prevalence of chronic diseases among older adults people (66), which often necessitates increased medical care services. For older adults individuals with chronic diseases, a well-developed community medical service environment tends to make them more inclined towards socialized older adults care. However, this impact is not significant for older adults individuals without chronic diseases. On the other hand, community psychological counselling services have a significant negative impact on the willingness of older adults individuals with chronic diseases to engage in social activities for older adults care. This implies that psychological counselling services may make older adults individuals with chronic diseases less inclined towards socialized older adults care. It is possible that older adults individuals with chronic diseases are more psychologically fragile due to their long-term illness and require more spiritual comfort. However, in Chinese culture, the care provided by the community is often considered less significant than the care received from one’s own children and family, which may contribute to this difference. Additionally, there is heterogeneity in the impact of entertainment venues on the willingness of older adults individuals who smoke to engage in social activities for older adults care within the community service environment. For older adults smokers, the positive impact of entertainment venues on their willingness to engage in social activities for older adults care is not significant. This can be attributed to the strong promotion and implementation of “smoking is prohibited in public places” and “smoking is harmful to health” policies. Their smoking behaviour is greatly restricted in entertainment venues, which may explain why these venues do not increase their willingness to engage in social activities for older adults care.

## 6 Conclusion

We aimed to investigate the influence of the community service environment on the willingness of older adults individuals in China to engage in social activities for older adults care. Previous studies have shown that various factors affect the retirement preferences of older adults individuals (54). In this study, we collected survey data to analyse and validate the impact of individual characteristics, family economic conditions, and support from children on the willingness of older adults individuals to seek socialized older adults care. Additionally, we found that the community, which is the primary living environment for older adults individuals, significantly influences their willingness to engage in older adults care, particularly in terms of the community service environment. Good community medical services, daily care services, and activity venues all increase the willingness of older adults individuals to participate in social activities for older adults care. However, the provision of psychological counselling services has the opposite effect. These findings provide valuable insights for improving the community environment and delivering higher-quality services. Importantly, due to the limitations of the questionnaire and sample, some conclusions of the study may not align with previous findings. In future studies, we will further explore the reasons underlying the impact of the community service environment on the willingness of older adults individuals to engage in social activities for older adults care, particularly why the provision of psychological counselling services does not increase their willingness to participate in such activities.

## Data availability statement

Publicly available datasets were analyzed in this study. This data can be found at: http://class.ruc.edu.cn/. Further inquiries can be sent to the corresponding author.

## Ethics statement

Ethical approval was not required for the study involving humans in accordance with the local legislation and institutional requirements. Written informed consent to participate in this study was not required from the participants or the participants' legal guardians/next of kin in accordance with the national legislation and the institutional requirements.

## Author contributions

DH: Formal analysis, Data curation, Writing – review & editing, Writing – original draft, Software, Methodology, Conceptualization. RF: Writing – review & editing, Software, Data curation. MS: Writing – review & editing, Software, Formal analysis.
